# Rarer than rare: managing an epiphrenic diverticulum in achalasia

**DOI:** 10.1055/a-2653-8710

**Published:** 2025-08-08

**Authors:** Giovanni Aldinio, Caterina Pelligra, Laura Cini, Marina Coletta, Beatrice Marinoni, Matteo Porta, Gian Eugenio Tontini

**Affiliations:** 1 9304 Department of Pathophysiology and Organ Transplantation, University of Milan, Milan, Italy; 2 9339 Gastroenterology and Endoscopy Unit, Fondazione IRCCS Caʼ Granda Ospedale Maggiore Policlinico, Milan, Italy; 3 9339 Department of Emergency Surgery, Fondazione IRCCS Caʼ Granda Ospedale Maggiore Policlinico, Milan, Italy

An epiphrenic diverticulum is an extraordinarily rare condition, occurring in approximately 1 per 500.000 people per year 1 . It is often associated with esophageal motility disorders (achalasia in 60% of cases) and, when large (i.e. >5 cm), undoubtedly causes symptoms such as dysphagia, regurgitation, weight loss, and aspiration pneumonia 1 2 . Malignant transformation, mostly into squamous cell carcinoma, occurs in about 0.6% of cases 3 . When a symptomatic diverticulum is associated with a motility disorder, subsequent management must address both conditions: minimally invasive surgery usually includes resection of the diverticulum, myotomy of the lower esophageal sphincter (LES), and an antireflux procedure 3 .

A 77-year-old man presented to the emergency room with progressive dysphagia and food regurgitation. Following two unsuccessful esophagogastroduodenoscopy (EGD) attempts at another hospital, a barium esophagogram showed a dilated esophagus with a prominent diverticulum above the LES ( Fig. 1 ). An EGD was eventually completed, revealing a markedly dilated, atonic esophagus filled with partially digested food, and a large epiphrenic diverticulum ( Video 1 ). The esophagogastric junction was passed with slight resistance using a 11.6-mm wide, high definition gastroscope (Pentax EG34-i10). A “Contents, Anatomy, Resistance, and Stasis” (CARS) score of 7 was highly suggestive of achalasia 4 . Type II achalasia was diagnosed via high resolution manometry (HRM), according to Chicago classification 4.0 ( Fig. 2 ).

**Fig. 1:**
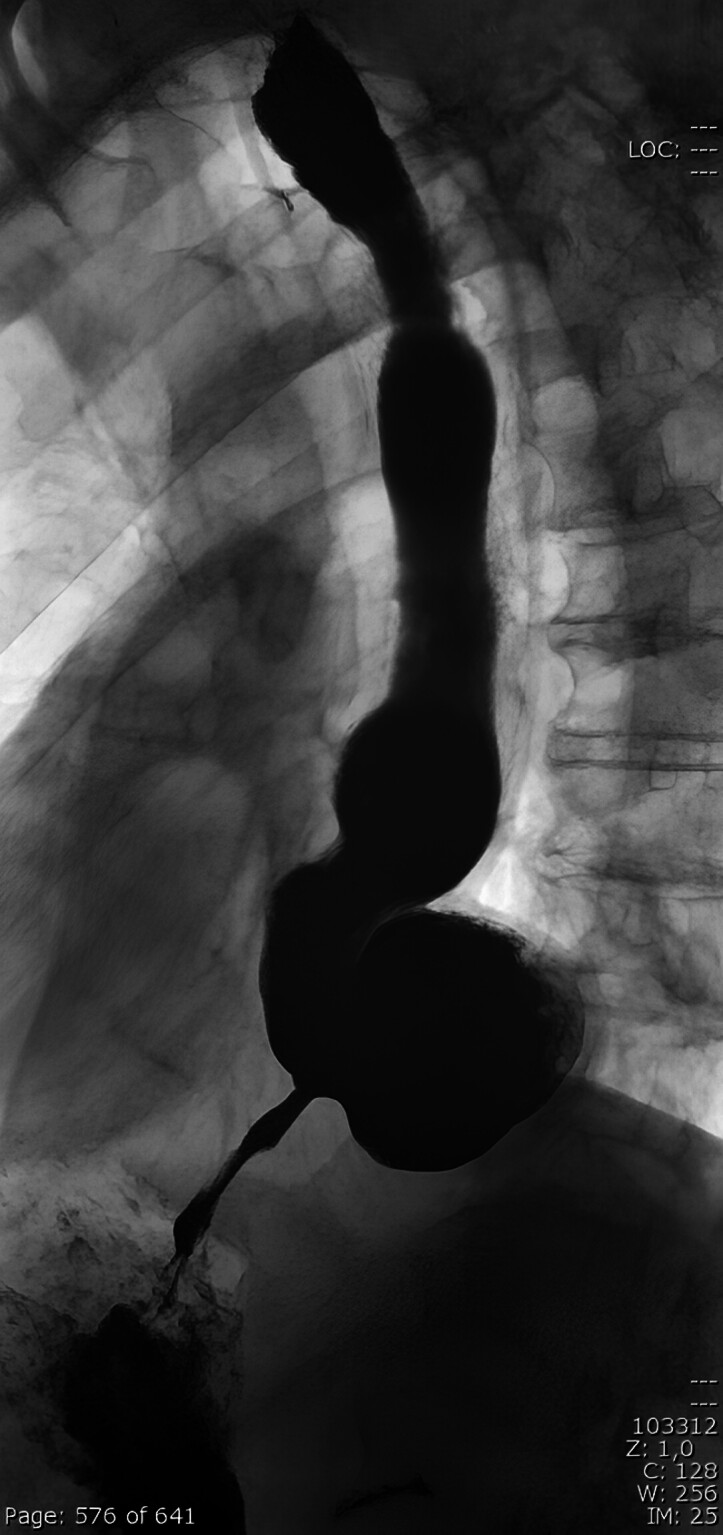
Initial barium esophagogram showing a dilated esophagus with a prominent diverticulum above the lower esophageal sphincter.

**Fig. 2:**
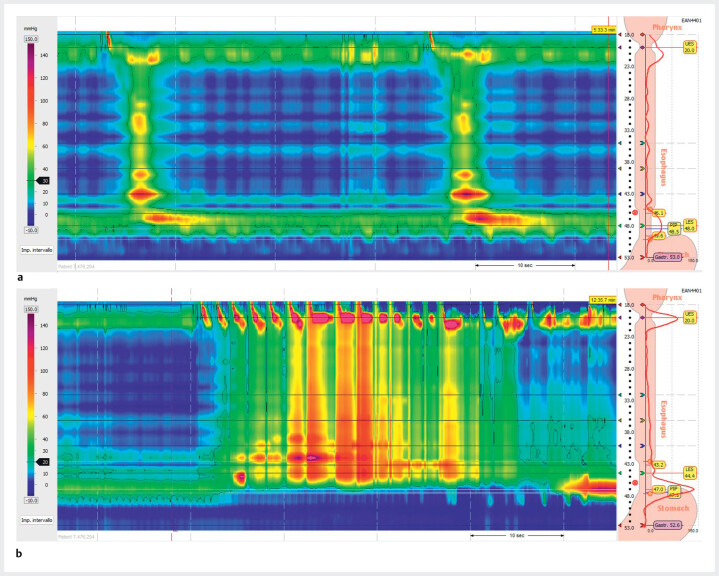
High resolution manometry showing the absence of lower esophageal sphincter (LES) relaxation ( **a** : single swallow, integrated relaxation pressure [IRP] = 32.5 mmHg; **b** : rapid drink challenge, IRP = 36.5 mmHg), with concomitant esophageal panpressurizations , which is compatible with type II achalasia.

Type II achalasia and a large epiphrenic diverticulum were diagnosed on barium esophagogram, esophagogastroduodenoscopy, high resolution manometry, and computed tomography before the patient underwent laparoscopic diverticulectomy, Heller myotomy, and Dor fundoplication.Video 1

A preoperative abdominal computed tomography scan confirmed dilatation of the proximal and mid esophagus and a 6 × 5-cm diverticulum ( Video 1 ). The patient underwent laparoscopic transhiatal diverticulectomy, Heller myotomy, and Dor fundoplication under intraoperative endoscopic guidance. No perioperative complications occurred. Histology was subsequently negative for malignancy. A postoperative contrast esophagogram showed no leaks and good transit, allowing oral feeding and discharge within a week. At 3-month follow-up, a barium esophagogram confirmed normal passage of barium and resolution of the diverticulum ( Fig. 3 ).

**Fig. 3:**
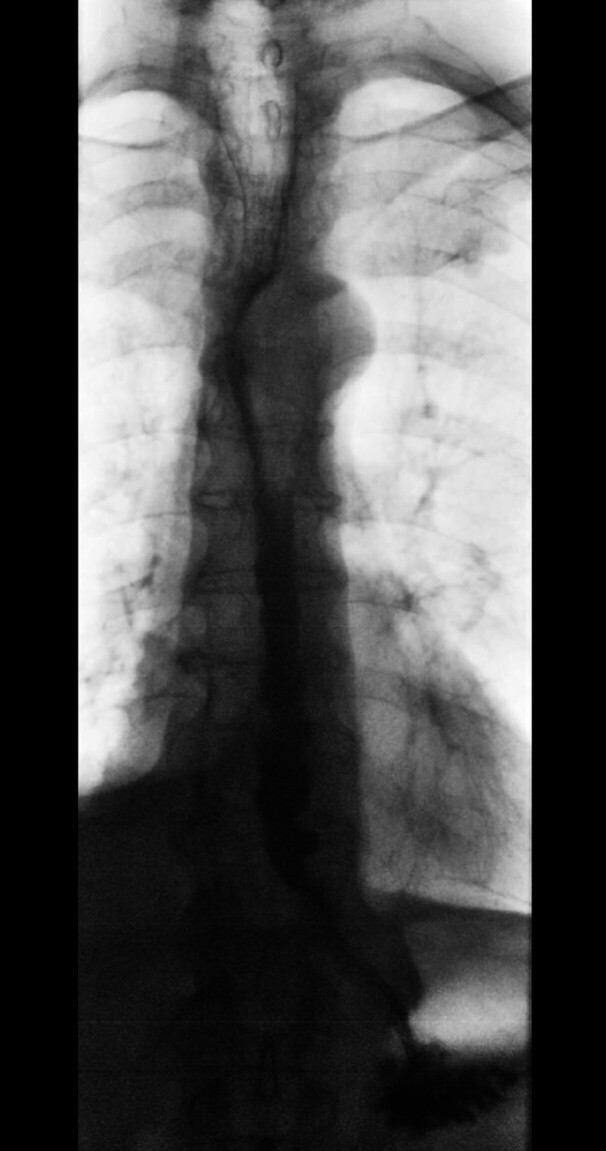
Repeat barium esophagogram at 3-month follow-up showing normal passage of barium and resolution of the diverticulum.

This case adds to the limited literature documenting epiphrenic diverticula in patients with achalasia, and their management 5 . A comprehensive diagnostic workup and a tailored surgical approach addressing both the diverticulum and the underlying motility disorder are essential to achieve optimal outcomes.

Endoscopy_UCTN_Code_CCL_1AB_2AC_3AF

## Notes

Endoscopy E-Videos https://eref.thieme.de/e-videos
*E-Videos* is an open access online section of the journal *Endoscopy* , reporting on interesting cases and new techniques in gastroenterological endoscopy. All papers include a high-quality video and are published with a Creative Commons CC-BY license. Endoscopy E-Videos qualify for HINARI discounts and waivers and eligibility is automatically checked during the submission process. We grant 100% waivers to articles whose corresponding authors are based in Group A countries and 50% waivers to those who are based in Group B countries as classified by Research4Life (see: https://www.research4life.org/access/eligibility/ ). This section has its own submission website at https://mc.manuscriptcentral.com/e-videos .
